# Prompting medical students to self–assess their learning needs during the ageing and health module: a mixed methods study

**DOI:** 10.1080/10872981.2019.1579558

**Published:** 2019-05-02

**Authors:** Grace Kennedy, Jennifer Nicola M. Rea, Irene Maeve Rea

**Affiliations:** a School of Medicine, Dentistry and Biomedical Science, Queens University Belfast, Belfast, UK; b Department of Primary Care, University College London, London, UK; c Stratified Medicine, C-TRIC, Biomedical Research Institute, The University of Ulster, Londonderry, UK; d Care of Elderly Medicine, Belfast Health and Social Care Trust, Belfast, UK

**Keywords:** Medical students, self-assessment, learning outcomes, quantitative and qualitative methods, ageing and health module

## Abstract

Understanding our learning needs is fundamental for safe, effective and knowledge-based medical practice and facilitates life-long learning. A mixed methods study investigated fourth-year medical students’ self-perceived understanding of their learning needs using 1] a visual scale, before and after a four-week module in Ageing and Health (A&H) and 2] through focus group discussions. During 2013–14 academic year, all students (252) were invited to use a Visual Analogue Scale (VAS) tool to self-assess their learning needs that were linked to Ageing and Health curriculum learning outcomes. Assenting students (197 at pre-self-assessment, 201 at post-assessment) returned anonymous Visual Analogue Scales, self-assessing history-taking skills, examination skills, knowledge of medication use, co-morbidity, nutritional and swallowing assessment responses, before and after the A&H module. Three student focus groups explored whether completion of the VAS self-assessment had prompted improved self-awareness of their learning needs. The VAS responses increased for each curriculum domain with significant differences between the pre-and post responses – for the student-year-group. Nutritional and swallowing knowledge showed the greatest improvement from a self-assessed low baseline at entry. Focus-group students generally viewed the VAS tool positively, and as an aid for prompting consideration of current and future clinical practice. Some students recognised that ‘*a need to be ready-for-work’* focused engaged learning; others demonstrated self-regulated learning through self-motivation and an action plan. The Visual Analogue Scale quantitative responses showed increased student-self-perceived learning for each curriculum domain at fourth-year completion of the A&H module, suggesting that prompting self-assessment had increased students’ knowledge and skills. Focus group students saw the VAS tool as useful for prompting awareness of their current and future learning needs. Additional educational strategies should be explored to enable all students to self-reflect and engage effectively on their learning needs, to gain the skills for the maintenance of professional medical competence.

**Abbreviations**: A&H: Ageing and Health Module; e-portfolio: an electronic version of an evidence portfolio, which allows medical students and graduates to reflect and document learning and competencies; F1: year1 of post-graduate medical clinical training; GMC: General Medical Council-the regulation organisation for maintaining standards for doctors in UK; Logbook: usually a written document which can be used to record procedures and attendance at clinics or case-based discussions and can be used to set learning outcomes and to structure teaching in clinical settings for medical students and doctors; PDP: personal development plan is used to plan future learning and skills needs for work and education with an plan for action/s outcome; SPSS: Statistical Package for the Social Sciences; VAS: Visual Analogue Scale is a visual method of describing an experience.

## Background

Self-directed learning is a core concept that is fundamental to adult education. In its simplest form, self-directed learning means that the learner exerts control over their own learning and directs strategies to manage their learning tasks. Garrison [1] elaborated a more complex model in which there is the integration of self-management (contextual control) together with self-monitoring (cognitive responsibility), and motivation (entering and task), as dimensions that reflect a meaningful and worthwhile approach to self-directed learning. In their consideration of self-assessment Eva & Regehr [2] suggest that the learner requires to recognise both their weaknesses and strengths, in a way that enables them to set challenging but reachable goals. Pisklakov et al., 2014 [3] in their review of self-evaluation and self-assessment in medical student and medical resident education was of the view that self-evaluation was the ability of learners to judge the quality of their work, based on evidence, for the purpose of self-improvement.

Self-assessment has been argued to enhance learning, including deep and lifelong learning [4]. In their study, Sharma et al. 2016 [5] examined the self-assessment by undergraduate medical students on their subsequent academic progress and concluded that self-assessment increased the interest and motivation of students leading to enhanced learning and better academic performance, and helping them in the development of critical skills for analysis of their own work. Sitzmann and colleagues [6] in their meta-analysis to assess the validity of self-assessments of knowledge in the broad area of education and workplace training, suggested that self-assessments of knowledge are moderately related to cognitive learning, motivation and satisfaction.

Student self-assessment has been studied previously [7–9] but is known to vary across individuals and context [2,10,11]. Although self-assessment is used to help learners identify their own learning needs, the available literature suggests there are conflicting results about reliability and validity [2,12,13]. Self-evaluation does not come naturally and requires training [14], with some evidence to show that learning and reflection were improved when learners practised self-assessment and received good feedback. Therefore, self-assessment has remained an important learning and educational developmental tool for both students and teachers [6].

The General Medical Council (GMC) UK, in its ‘*Assessment in Undergraduate Medical Education; Overview a clear strategy*’ (2011), advised that educational strategy should cover formative as well as summative assessment, and that *‘Students should become accustomed to seeking maximum benefit from feedback, self-assessment, reflection and the development of lifelong learning skills’* [15]. The current GMC consultation ‘*About the Outcomes for Graduates 2017ʹ* (GMC 2017) [16], again advises that medical students should commit to lifelong learning, and take responsibility for their own learning.

In order to further explore the concept of self-assessment, we invited medical students at Queen’s University Belfast to assess their self-perceived learning needs during their fourth-year clinical attachment in Ageing and Health, by using a Visual Analogue Scale (VAS) [17]. The Visual Analogue Scale (VAS) used in this study, measured a characteristic – self-perceived learning need- that cannot be easily quantified by ordinal scales, such as the Likert scale. The VAS has been used widely in clinical practice and in nursing teaching programmes [17–19]. The student anonymous returns allowed us to evaluate any change in self-perceived learning and skills for six main curriculum domains in the Ageing and Health (A&H) module. As an adjunct to the quantitative analysis of the Visual Analogue Scale (VAS) returns, three student focus groups were invited to discuss issues around student understanding of the use of self-perceived assessment as a means of improving insight into their current in-module learning needs and for their future medical practice.

## Methods

### Setting

We conducted the study at Queens University Medical School, which offers a five-year curriculum with clinical medicine integrated from year one. The clinical curriculum has a modular structure; the sequence of the modules is identical for all students. The Ageing and Health Module (A&H) in the fourth year of the clinical medicine, comprises four weeks of clinical Elderly Care Medicine the first week of lectures, seminars, case-based study, and three weeks of clinical teaching sessions under the supervision of speciality consultants. Six groups of students (40–42 approximately) rotate through the curriculum and through Ageing and Health module in consecutive blocks -A, B, C, D, E and F during the academic year.

### Participants

During 2013–14 academic year all medical students entering the Ageing and Health module were invited to participate in a teaching evaluation of their self-perceived learning needs, for six areas of the curriculum learning outcomes with respect to elderly patients – history-taking skills, examination skills, medication use, co-morbidity, and nutritional and swallowing assessments. There were no selection or exclusion criteria. Participation was voluntary and anonymous students assenting to participate returned completed Visual Analogue Scale (VAS) responses, at the beginning and end of their A&H Module.

### Visual Analogue Scale tool (VAS)

**Figure 1.**
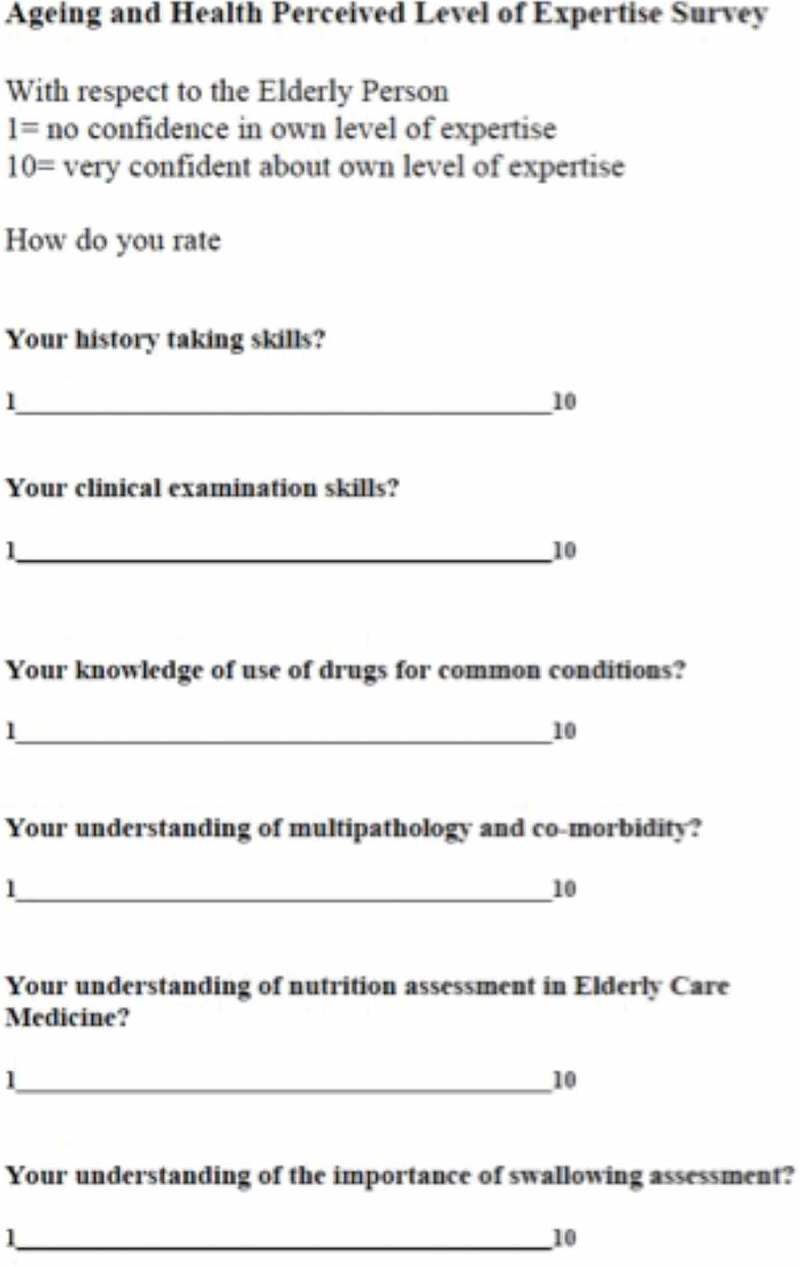
The Visual Analogue Scale. The Visual Analogue scale (VAS) is a graphic scale in which the respondent places a mark at a point along a line, with endpoints of 1 and 10 [8], with 1 representing very little knowledge/skills and 10 representing very good knowledge/skills. The VAS tool was completed for 6 domains in the A&H curriculum

The Visual Analogue scales (VAS) [17,18] is a graphic scale in which the respondent places a mark at a point along a line, with endpoints of 1 and 10 [18], with 1 representing very little knowledge or skills and 10 representing very good knowledge or skills. The student mark on the VAS scale was measured from the endpoint 1 and this distance calculated arithmetically as a percentage of the total measurement between the endpoints 1 and 10. The percentage was changed to a score out of 10. The VAS tool scale was completed for the six clinical domains in the A&H curriculum (Figure 1).

### Quantitative statistical analyses

The anonymous pre- and post VAS responses were measured with responses calculated out of 10 and analysed during a 6-week summer research studentship (GK). Comparison of pre- and post-module responses were made for the six knowledge and clinical domains, for the whole 2013–14 student year group and for the six individual blocks of students for A&H module, using two-tailed unpaired t-tests and non-parametric tests Mann–Whitney U and Kruskall Wallis as appropriate and SPSS analysis. A p-value <0.05 considered significant. Data sets are available at https://figshare.com/s/5469e44e08092002e41c. Doi: 10.6084/m9.figshare.7294922

### Focus groups and qualitative analysis

A general invite for interested students (4–8) in the academic year 2013–14 was made for focus group participation. Three focus groups took place – the first focus group early in academic year 2013–14, the second group late in the academic year 2013–14, and the third group early in the academic year 2014–15. Participating students gave written consent to take part in a semi-structured discussion facilitated by student researcher (GK) who had appropriate training and experience. Students were advised that the session (~30 min) would be recorded and transcribed but that no contributor would be identified in person. All focus groups used the same semi-structured interview (Table 1), but the second and third focus groups, used in addition, the thematic outputs from the first focus group, to further refine and probe the emerging thematic issues towards saturation. The transcribed discussions were read and re-read individually by each of the three authors and grounded theory qualitative methods [20] were used to establish emerging themes and linked concepts. Focus group output is available at https://figshare.com/s/5469e44e08092002e41c. Doi: 10.6084/m9.figshare.7294922.

**Table 1.** Focus group semi-structured questions.

The focus group will take the form of an opening introductory question followed by 4–6 semi-structured questions.
What did you feel about using the Visual Likert Scale? Did you think that using the Visual Likert scale helped you understand your learning needs in the Ageing and Health Module **at the beginning** of the Ageing and Health Module? Did you think that using the Visual Likert scale helped you understand about your learning needs in Ageing and Health Module **at the end** of the Ageing and Health Module? Did you think that using the Visual Likert scale and completing it **helped you focus on what you had learned** or where you had gaps in your learning in Ageing and Health? Do you think that assessing your own learning needs might link with setting up a **personal development plan (PDP)** for your learning needs and in use of e-portfolios? What about the importance of assessing your own learning needs as a preparation for life-long learning in your **future career in Medicine**?

### Ethical approval and consent to participate

Ethical approval for the focus groups was obtained from the Joint Research Ethics Committee, Queens University of Belfast, School of Medicine, Dentistry and Biomedical Science (Ref: 14.19.v2; Ref: 14.45). Students were given Information leaflets and assenting students provided written consent. The Visual Analogue Scale questionnaire within the project was part of a curriculum development of the teaching course providing the students with the opportunity to develop a core life-long skill, and viewed under the remit of the Joint Research Ethics Committee (Ref: 14.19v2).

## Results

### Visual Analogues Scale (VAS) analysis

There were a total of 398 anonymous completed VAS returns for self-perceived learning needs for the 2013–14 year group of medical students-197 at pre-self-assessment and 201 at post-assessment after completion of the A&H module. This represented an approximate 80% return from the 250 students who commenced fourth year medical studies. The numbers for completed and returned VAS scores in Groups A, B, C, D, E and F were as follows; Group A (33 pre- and 36 post-), Group B (36 pre -and 36 post-), Group C (37 pre-and 32 post-), Group D (pre-28 and post −27), Group E (pre 26 -and post-32) and F (pre −38 and post-38). No information was available for the graduate or sex status of the fourth year medical students who returned the VAS tool, which was anonymous.

### Pre- and post-Module Visual Analogue Scales (VAS) comparisons for the A&H clinical domains for the total student year

Table 2 shows the mean, variance, standard deviation and standard error for the pre- and post-aggregated VAS scores for the Total Student Year for each of the six A&H clinical domains. There are increases in history taking skills, examination skills, medication use, co-morbidity, nutritional assessment and swallowing assessment in the post-student compared to the pre-student  scores which are highly significant when compared by unpaired two-tailed student t-test.

**Table 2.** Comparison of Visual Analogue Scale scores for pre- and post- self-perceived assessment of learning needs for total student year group for six clinical domains.

	Pre-				Post-				t-test
Clinical Area	Mean score	Var	SD	SE	Mean	Var	SD	SE	p-value
History-taking skills	**6.67**	1.69	1.30	0.09	**7.57**	1.24	1.11	0.08	P < 0.0001
Examination skills	**5.89**	2.01	1.43	0.10	**7.22**	1.35	1.16	0.08	P < 0001
Medication use	**5.68**	2.46	1.57	0.11	**7.03**	1.66	1.29	0.09	P < 0001
Co-morbidity	**5.40**	1.98	1.41	0.10	**7.09**	1.28	1.13	0.08	P < 0.0001
Nutritional assessment	**3.74**	3.35	1.83	0.13	**6.49**	2.6	1.61	0.11	P < 0.0001
Swallowing assessment	**4.35**	4.59	2.14	0.15	**7.72**	2.50	1.58	0.11	P < 0.0001

Means in **Bold**; Var, Variance; SD Standard deviation; SE standard Error; P < 0.01 significant, Comparison with unpaired two-tailed Student t-test

**Figure 2.**
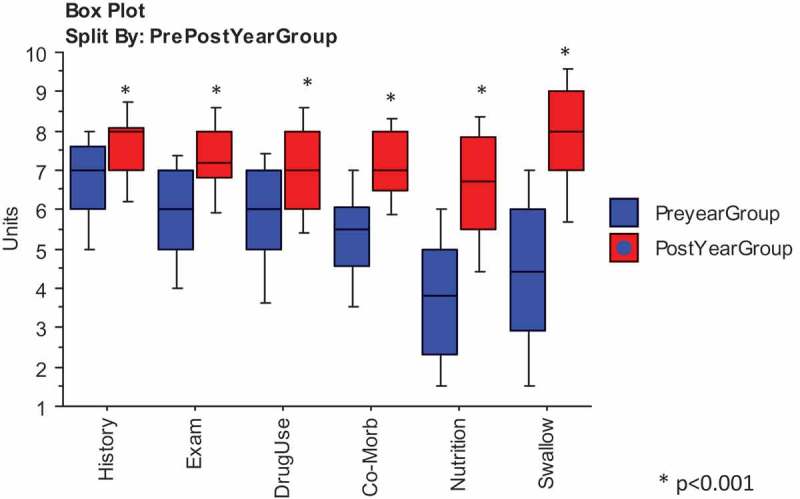
Comparisons visual analogue scores for total year student year pre- and post-scores for self-perceived assessment of learning needs. Box plots for comparison of pre- and post-Visual Analogue Scale (VAS) responses for self-perceived Assessment of Learning Needs in each of six curriculum domains in Ageing and Health, for the total student Year, showing medians and 25% and 75% values.Analysis by Mann–Whitney U; P values <0.001 considered significant.*Significant differences between Pre-and Post-measurements for each curriculum domain.

Figure 2 demonstrates significant increases in the post-VAS scores compared to the pre-scores for self-perceived learning needs for the aggregated whole year group, shown as box-plots, medians and 25% and 75% ranges, using Mann Whitney U test statistic. Both nutritional and swallowing assessments, comparatively new areas of clinical knowledge introduced to the fourth year students in the A&H module showed an increase of approximately three points in the post-VAS score. There was also a wider range between pre- and post-VAS scores with many students showing low pre-module scores in comparison to more familiar areas of history taking and examination skills (Figure 2).

### Pre- and post-Module Visual Analogue Scales (VAS) comparisons for A&H clinical domains for the student class groups A, B, C, D, E and F

**Figure 3.**
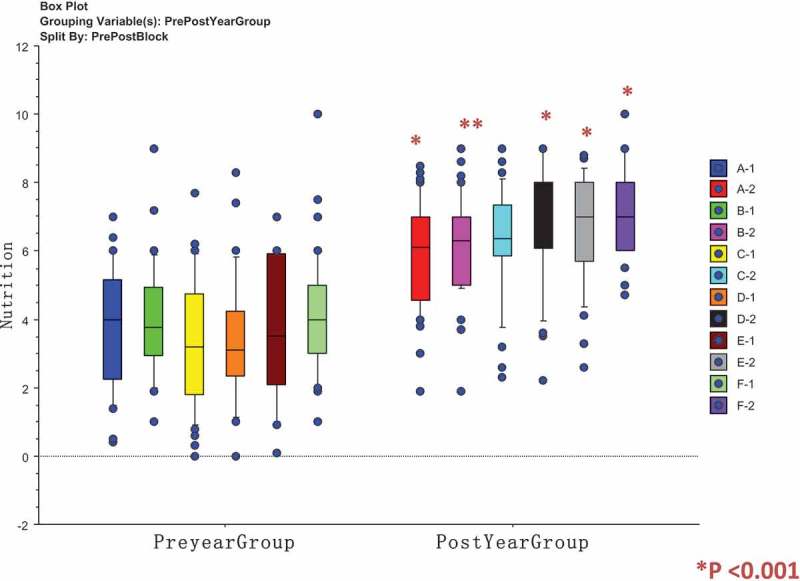
Comparisons of Visual Analogue Scale scores for student group A, B, C, D, E, and F showing pre- and post-VAS scores for self-perceived learning needs for nutrition. Box plots for comparison of pre- and post-Visual Analogue Scale (VAS) responses for self-perceived Assessment of Learning Needs in each of six curriculum domains in Ageing and Health, showing medians and 25% and 75% values; Analysis by Mann–Whitney U; P values <0.001 significant; Pre-groups are A-1, B-1, C-1, D-1, E-1 and F-1; Post – Groups are A-2, B-2, C-2, D-2, E-2 and F-2.

The means for pre- and post-VAS scores for each of the six student class groups A, B, C, D, E and F and for each of the six curriculum domains were analysed by unpaired two-sided t-test are shown in Table 3. The post-student self-assessment VAS scores demonstrate increases in self-rating self-assessment response for post-VAS for each student class group for the clinical domains – examination skills, medication use, comorbidity and nutrition and swallowing assessment that are significant (Table 3). The history taking skills for student class groups A and D did not show a change post-self-assessment from pre-self-assessment scores that was significant, and it is considered that many students in fourth Year medicine would have already gained a good standard of history taking skills when commencing the A&H module. Figure 3 shows box-plots, medians and 25% and 75% ranges for the individual student class groups (ABCDEF) for nutrition, a clinical skill where students showed a three-point post-self-assessment score increase. Pre-VAS self-assessment scores are shown as (A-1, B-2, C-1, D-1, E-1, F-1) and post-VAS scores as (A-2, B-2, C-2, D-2, E-2, F-2) and demonstrate a significant increase across all the class groups, using Mann Whitney U test statistic.

**Table 3.** Comparison of pre- and post-Visual Analogue Scale (VAS) scores for student groups A-D for six domains of the curriculum.

Clinical area	Group A	Group B	Group C	Group D	Group E	Group F
History-taking skills	6.52[0.24] **7.02**[0.27] p = 0.165	6.63[0.18] **7.58**[0.17] p = 0.0002	6.77[0.19] **7.76**[0.16] p = 0002	6.73[0.29] **7.30**[0.25] p = 0.149	6.40[0.31] 7.79[0.12] p < 0001	6.87[0.21] **7.92**[0.12] p < 0001
Examination skills	5.82[0.25] **6.86**[0.24] p = 004	5.90[0.22] **7.11**[0.18] p < 0001	5.83[0.26] **7.39**[0,16] p < 0001	5.81[0.27] **6.95**[0.25] p = 003	5.89[0.34] **7.37**[0.19] p = 0002	6.04[0.21] **7.60**[0.17] p < 0001
Medication use	5.03[0.30] **6.52**[0.27] p = 0004	5.86[0.22] **6.96**[0.20] p = 0004	5.84[0.29] **7.00**[0.24] p = 0032	5.94[0.31] **7.13**[0.24] p = 0038	5.44[0.29] **7.06**[0.24] p < 0001	5.87[0.23] **7.52**[0.17] p < 0001
Co-morbidity	5.12[0.23] **6.67**[0.23] p < 0001	5.4[0.21] **7.0[**0.17] p < 0001	5.48[0.27] **6.92**[0.22] p < 0001	5.70[0.29] **7.21**[0.21] p < 0001	5.12[0.29] **7.18**[0.15] p < 0001	5.54[0.21] **7.55**[0.16] p < 0001
Nutritional assessment	3.84[0.31] **5.84**[0.27] p < 0001	3.82[0.28] **6.19**[0.25] p < 0001	3.32[0.31] **6.27**[0.29] p < 0001	3.46[0.36] **6.87[**0.33] p < 0001	3.61[0.40] **6.56**[0.28] p < 0001	4.26[0.29] **7.22**[0.22] p < 0001
Swallowing assessment	3.89[0.36] **7.01**[0.31] p < 0001	4.09[0.31] **7.41**[0.26] p < 0001	4.10[0.35] **7.67**[0.29] p < 0001	4.59[0.49] **8.00**[0.24] p < 0001	3.93[0.37] **7.79**[0.25] p < 0001	5.37[0.34] **8.46**[0.20] p < 0001

Means and standard Errors []; **Bold** = Post-VAS Score; Comparisons of pre and post-VAS scores for consecutive Student Blocks A-D during the academic year by unpaired two-tailed Student t-test; **p < 0.01 significant. ns = non-significant.

### Focus groups and themes

Six, 4^th^ year medical students participated in the first focus group, five, 4^th^/5^th^ year medical students in the second focus group, and four 5^th^ year medical students in the third focus group. All participating students were recruited during the 2013–14 academic year, but because of irreconcilable timetabling for student and facilitator availability, the third focus group took place when students entered 5^th^ year medicine.

## Findings

**Figure 4.**
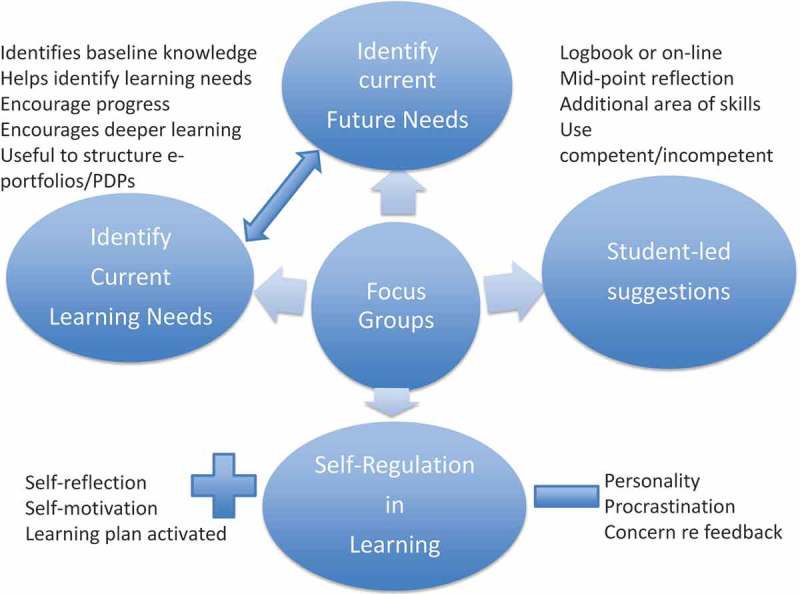
Four themes with sub-themes emerging from the focus groups discussions about student self-assessment of learning needs. The four themes are: 1] Identifying Current Learning Needs; 2] Future Use in Identifying Learning Needs; 3] Student-Led Suggestions in VAS Use; 4] Learning Environment Issues in Use of VAS; 5] Self-Regulation in Learning.

The four key themes emerging from the three focus groups with respect to the students’ understanding and use of the Visual Analogue Scale as an adjunct to their learning are outlined under the main headings in Figure 4.

**The 4 themes are: 1] Identifying Current Learning Needs; 2] Future Use in Identifying Learning Needs; 3] Student-Led Suggestions in VAS Use; 4] Self-Regulation in Learning.**

**Theme 1 Identifying Current Learning Needs**

**1.1 Improved Awareness of Learning Needs**

Students’ comments on their use of the VAS pre- and post- the A&H module shaped this theme. **Before** the module a student advised ‘*it* (the VAS) *was quite good to help structure and give… areas to think about where your needs were’*, with another adding it was a ‘*good indicator of baseline knowledge’*. **After** the module, a cluster of comments reflected that VAS had helped identify learning needs still present, as in ‘*its good to know what you are good at and what you need to practise*’.

**1.2 Easy to Use**

In this **sub-theme** students generally saw the VAS as easy to use, one student advising ‘*Yeah it was grand; its easy to use, very straight forward’*.

**Theme 2 Future Learning Needs**

**2.1 Useful to structure learning in e-portfolio and Personal Development Plans (PDPs)**

In this **second them**e, students commented on several aspects of their future learning. One student said ‘*I think (VAS) would give you some structure for setting out your* PDPs [personal development plan]’, and in considering e-portfolio use, a student commented ‘*If you were* (to) *have those kind of scales and it was more focused, the e-portfolio would be more beneficial* ‘.

**2.2 Improving deeper levels of Understanding**

Students could see beyond 4^th^ year undergraduate studies and to their future medical practice commenting ‘*I think I knew in the future, we’d have to write reflections*, [so] *I actually made a list of stuff that I need to learn how to do*’. There was recognition that skills should not remain static as in ‘*It’s all about improving our skills and improving’.*

**2.3 Focused Learning for entering Workplace**

Students recognised a different learning approach between 4^th^ and 5^th^ year as the reality of being a doctor in charge of patients became closer, saying *‘I supposed we’re very F1-focused now* [year1 post-graduate medical training] *…whereas in 4^th^ year, I don’t know ….. that I’d have got my head into that space*’. Another student volunteered -‘*It [*VAS and self-assessment*] is something I’m only really seeing the importance of n*ow… *now that we’re actually faced with the world of work*’.

**Theme 3 Student-Led Suggestions for VAS Use**

**3.1 Logbook or on-line modification**

The students were ready with suggestions to improve the VAS tool and thought an on-line or logbook version for self-assessment should be considered -‘*if you could do it on-line and* [if] *you did it at the start and at the end of a six week module and then it was able to give feedback’.*

**3.2 Mid-Module Reflection Opportunity**

A **related sub-theme** suggested that ‘*it* [VAS] *should have a mid-point reflection-then you have the educational opportunities that you can tailor to what you need’*.

**3.3 Binary competent/non-competent for some Clinical Skills**

A comparison of the binary terms ‘competent’ or ‘non-competent’ rather than the VAS scale was discussed as perhaps more useful for assessing clinical knowledge, as in-‘*I would be more in favour of “competent” or “not competent” rather than a Likert scale’*. Another student agreed with this idea but also recognised that ‘different skills should be assessed differently as in ‘(For) *a nutritional assessment…that’s a ‘yes’ or ‘no*’, but ‘*history-taking is so varied; it’s not an assessment where it’s either” yes” or ‘no*’.

**Theme 4 Self-Regulation**

**4.1 Self-Reflection**

In this **theme**, some students spoke of recognising their strengths and weaknesses as in *‘there’s so much to know that it’s really important to be able to identify where you are weaker*’. Recognising that each person will reflect differently on their own learning needs, others commented ‘*I think it depends how critical you are of yourself. So one person’s nine could be another person’s five*’.

**4.2 Self-Motivation**

Motivation was recognised as important in addressing learning needs, with a student explaining ‘*the VAS probably does make you think* “*I’d need to have a look at that” but whether or not you have the self-drive to do that is another question*’. A number of students acknowledged that personality was part of the self-motivation response with one student saying ‘*I think it depends on the person; some people are going to care about it more than others. It’s really subjective*’.

**4.3 Acting on Learning Needs**

There were different approaches to acting on learning needs. One student told how he/she had used the VAS during the A&H module saying *‘there was a question about your drug knowledge so I knew I needed to concentrate on that. During attachment I was looking at the* [drug*] kardex a lot’*. Another recognised the role of personality as in *‘You have to make that decision whether you want to improve on that area’*.

## Discussion

In this study of self-perceived assessment of learning needs, we found that the class year of 4^th^ year medical students’ evaluation of their self-assessed learning needs, showed increases for each of the six core curriculum domains in Ageing and Health (A&H) that were significant, when comparing the pre- and post-module responses, using the visual analogue scale (VAS).

A number of studies have looked at ‘*before and after*’ assessment in teaching programmes. In a longitudinal study, Arnold et al. [21], asked medical students to complete self-evaluation at the close of each rotation during a six-year curriculum. The authors found that both students’ self-evaluations and faculty members’ ratings of students’ performance increased year by year, but conversely the relationship between both decreased each year. Despite these dissonant findings, both groups considered that self-evaluation encouraged learning and professional development. In the pre- and post- self-assessment of learning outcomes in one hundred and forty-five, fourth-year students at Göttingen Medical School study, Schiekirka et al. [2013] reported that there was good agreement between the performance-gain derived from the formative examination data and performance gain derived from the student self-assessment data in the 33 specific learning objectives, in a cardiorespiratory module [22]. In a Stop Think approach to encourage self-assessment of learning, a research study invited students to rate their feeling of difficulty before and after completing an anatomy or physiology concept map exercise [23]. The differences in the results between the students’ estimation of their pre- and post estimated difficulty score correlated with students’ objective-class examination scores, suggesting that this method appeared to be successful in stimulating students to self-assess their learning activity. In other research such as a small study of videotaped communication skills, there was a lack of concordance between undergraduate medical students’ own assessment of self-efficacy and trained observers’ assessment at two different monitoring points [24]. Similarly, Gruppen and colleagues found a very low correlation (0.25) between the observer and self-assessed student rating in 10 areas of curriculum [25].

The Visual Analogue Scale (VAS) used in this study, measures a characteristic – self-perceived learning need- that cannot be easily quantified by ordinal scales, such as the Likert scale. However, it could be argued that translating the change in students’ self-perceived learning need after the Ageing and Health module, into a numeric evaluation may or may not be a true reflection of the metacognitive nature of their learning. Against this suggestion, the VAS has been used widely in clinical practice [17,18], in social and behavioural research [26], in business studies [27], in pain assessment medicine [28,29] and in nursing teaching programmes [30]. A benefit of this type of visual analogue scale as opposed to a Likert scale with 5 or 7 degrees of agree/not agree, is that students can visually estimate their learning needs and later comparisons are considered a truer reflection of change. Celenza & Rodgers [19] described the VAS tool as reliable and a valid alternative to a Likert-type scale (LTS), in their evaluation of perceptions of a bedside-clinical-teaching programme. In reviewing the benefits and shortcomings of the VAS in measuring clinical phenomena, Wewers & Lowe [31] argued that the VAS was of most value when comparing change within individuals or groups over time, but was likely of less value for comparisons at one time-point. In the present study, the VAS tool was used to produce interval data between the pre-and post-estimations of student-perceived-learning needs for the Student Year or Student class-group, which according to these authors is more likely of greater value and reliability [31]. Similarly, in a commentary on self-assessment, Lam (2009) argued that aggregated self-assessments were likely more useful in comparison data [32].

But irrespective of which method, scale or analysis is used for self-assessment, most studies suggest that it is difficult to self-assess accurately. In investigating undergraduate students’ self-assessment ability in comparison with their instructor’s judgement, both Mattheos et al. [33], and Rees & Shepherd [34] reported that students tended to overestimate their competence compared to teachers. Similarly, Davis and colleagues [35] in a meta-analysis comparing the accuracy of physician self-assessment compared to externally assessed competencies found that physicians had a limited ability to accurately self-assess. A number of studies have reported that females perform better than males in observed examinations although their self-assessment scores tended to be lower [24,33]. There is limited evidence to suggest that students improve their self-assessment skill over time [21,33,36,37], though self-assessment is considered a learnable skill [38].

## What did the focus groups tell about student self-assessment?

In the **first and second themes**, students saw the VAS tool as a potential aid for identifying their current and future self-directed learning needs, and this was encouraging. However, a few negative comments suggested that some students had no apparent understanding of the importance of developing self-directed learning skills. But as students reached final year, the need to be ready-for-work focused their learning, similar to the work-place-imminent–learning behaviour recognised by Billet [39].

A further theme reflected students’ readiness to make suggestions for improvements to the VAS. They suggested the VAS for self-assessment should be incorporated into an online version of the logbook to support self-reflection. These important student-led suggestions for the VAS have led to developmental changes being incorporated into the A&H logbook with monitoring of learning outcomes. Students also needed to be reassured that self-assessment was being used as a student self-development skill for workplace learning and not for use by Faculty [40,41].

Students demonstrated different learning styles from some able to self-regulate their learning to others who seemed less likely to act on their weaknesses. These thematic outputs prompt teachers, clinicians and educators to consider the many different student learning-styles, the complex and changing clinical environments in which students are expected to learn [42] and the embodied tensions of self-assessment- ‘*between wanting to know and afraid to know’* - [43,44].

## Strengths

This was a mixed methods study providing useful insights from using a combined quantitative and qualitative approach. We used six of the main learning objectives from the A&H course for the self-assessment study so covering the most important clinical and knowledge learning outcomes of the four-week A&H attachment. The study was simple to set up, easy to implement and was not time-consuming for either participating students or the research supervisor. There was a high student response rate in study and we did not use incentives to encourage students to take part in the study, hence reducing bias. This particular method is valuable as it did not require individual labelling of students and was conducted completely anonymously. The VAS took initial student performance into account and the performance gain regarding specific objectives was calculated from aggregated data at the group level and did not require extensive pre-and post-testing.

The qualitative aspect of the project gave some encouraging insights into the students’ engagement, use and value of the process of self-assessment. It allowed the students’ views to be heard and student-led ideas to become incorporated in the processes of self-assessment in teaching and learning outcomes in the A&H module. The whole process became a tool for empowering students by giving them a sense of shared decision-making in course improvement and demonstrating that self-assessment could enhance the quality of their learning as students and thorough out their medical careers.

## Limitations

The study’s findings have a number of limitations. Since participation was voluntary and anonymous, only students who completed the VAS were included in the quantitative analysis and the results are presented as aggregated student-year and the rotating six student-group results. It is not known whether some of the same students joined the focus groups. The emerging results and themes may therefore not be representative of the whole year group and so the outcomes should be considered exploratory. The effect of either student gender or graduate status could not be assessed because student responses were anonymous. The study involves only one university and may not have wider validity. In addition, the focus groups were relatively small, although numbers alone should not argue against the quality of the output obtained.

The observation from other studies that increased scores or performance gains did not necessarily mean that actual learning had taken place, equally applies to our research findings. In addition, the body of evidence showing that students tend to overestimate their competence in relation to the judgement of their instructors and that increased self-confidence can compromise patient safety is highly relevant [21,33–35] and is also applicable.

## Summary comments

Self-assessment of clinical knowledge and accuracy of skill performance is considered essential to the practice of medicine and self-directed life-long learning and best practice should be encouraged in order to improve and advance the skill [40,45]. Medical practitioners are responsible for their own learning, through self-assessment (reflection, self-monitoring, external information seeking) [46].

Undergraduate medical students or junior doctors in-training often do not take time to actively think about their learning needs before they enter a new learning module or clinical environment rotation. Here we have used a simple VAS tool to aid students to self-assess their learning their needs. We demonstrated a significant ‘performance gain’ in their self-perceived knowledge and skills after the Ageing and Health module with evidence that the students had used self-assessment to improve their knowledge and skills. The qualitative aspects of the project demonstrated that students engaged in the process and saw self-assessment as beneficial. It also gave students a sense of shared decision-making and allowed them to advance student-led suggestions for improvements in the A&H module. Finally focus group discussions demonstrated that students recognised how self-assessment was a tool that could be used to improve their current and future medical knowledge and skills.

## Conclusions

- Students’ self-perceived knowledge and skills increased in domains linked to learning outcomes after the A&H module.
- In focus groups, students generally viewed the VAS tool positively, and as an aid for identifying current and future learning needs.
- New educational strategies should be developed in the Medical School Curriculum so that all students can maximise their self-reflective and self-assessment skills.

## Data Availability

All data generated or analysed during this study are included in this published article [an in supplementary information files-https://figshare.com/s/5469e44e08092002e41c. Doi: 10.6084/m9.figshare.7294922].

## References

[1] Garrison DR. Self-Directed learning: toward a comprehensive model. Adult Educ Q Fall. 1997;48:18–12.

[2] Eva KW, Regehr G Self-assessment in the health professions: a reformulation and research agenda. Acad Med. 2005;80:S46–S54. 16199457 10.1097/00001888-200510001-00015

[3] Pisklakov S, Rimal J, McGuirt S Role of self-evaluation and self-assessment in medical student and medical resident education. Br J Educ Soc Beh Sci. 2014;4:1–9.

[4] Andrade H, Du Y Student responses to criteria-referenced self-assessment. Assess Eval Higher Educ. 2007;32(2):159–181.

[5] Sharma R, Jain A, Gupta N, et al Impact of self-assessment by students on their learning. Int J App Basic Med Res. 2016;6:226–229. 10.4103/2229-516X.186961 PMC4979309 27563593

[6] Sitzmann T, Ely K, Brown KG, et al Self-assessment of knowledge: a cognitive learning or affective measure? Acad Manage Learn Educ. 2010;9:2169–2191.

[7] Colthart I, Bagnall G, Evans A, et al The effectiveness of self-assessment on the identification of learner needs, learner activity, and impact on clinical practice: BEME guide no 10. Med Teach. 2008;30:124–145. 18464136 10.1080/01421590701881699

[8] Falchikov N, Boud D Student self-assessment in higher education: a meta-analysis. Rev Educ Res. 1989;59:395–430.

[9] Davis DA, Mazmanian PE, Fordis M, et al Accuracy of physician selfassessment compared with observed measures of competence: a systematic review. JAMA. 2006;296:1094–1102. 16954489 10.1001/jama.296.9.1094

[10] Regehr G, Hodges B, Tiberius R, et al Measuring self-assessment skills: an innovative relative ranking model. Acad Med. 1996;71(10 suppl):S52–S54. 10.1097/00001888-199610000-00043 8940934

[11] Fitzgerald JT, White CB, Gruppen LD A longitudinal study of self-assessment accuracy. Med Educ. 2003;37:645–649. 12834423 10.1046/j.1365-2923.2003.01567.x

[12] Gordon MJ A review of the validity and accuracy of self-assessments in health professions training. Acad Med. 1991;66:762–769. 1750956 10.1097/00001888-199112000-00012

[13] Ward M, Gruppen L, Regehr G Measuring self-assessment: current state of the art. Adv Health Sci Educ Theory Pract. 2002;7:63–80. 11912336 10.1023/a:1014585522084

[14] Fredericksen J, Collins A A systems approach to educational testing. Educ Researcher. 1989;18:27–32.

[15] GMC 2011 Assessment in undergraduate medical education;overview a clear strategy. [cited 2019 112] Availabe from: https://www.gmc-uk.org/Assessment_in_undergraduate_medical_education___guidance_0815.pdf_56439668.pdf

[16] GMC 2017 About the outcomes for graduates GMC2017. [cited 2019 112] Availabe from: https://www.gmc-uk.org/Outcomes_for_graduates_2017_v0.22_final_for_consultation.pdf_72053747.pdf

[17] Guyatt GH, Townsend M, Berman LB, et al A comparison of Likert and visual analogue scales for measuring change in function. J Chron Dis. 1987;40:1129–1133. 3680471 10.1016/0021-9681(87)90080-4

[18] Gift AG Validation of a vertical visual analogue scale as a measure of clinical dyspnea. Rehabil Nurs. 1989;14(6):323–325. 2813949 10.1002/j.2048-7940.1989.tb01129.x

[19] Celenza A, Rogers IR Comparison of visual analogue and Likert scales in evaluation of an emergency department bedside teaching programme. Emerg Med Australas. 2011;23:68–75. 21284816 10.1111/j.1742-6723.2010.01352.x

[20] Glaser BG, Strauss AL The discovery of grounded theory: strategies for qualitative research. New York: Aldine de Gruyter; 1967.

[21] Arnold L, Willoughby TL, Calkins EV Self-evaluation in undergraduate medical education: a longitudinal perspective. J Med Educ. 1985;60(1):21–28. 3965720 10.1097/00001888-198501000-00004

[22] Schiekirka S, Reinhardt D, Beibarth T, et al Estimating learning outcomes from pre and posttest student self-assessments: a longitudinal study. Acad Med. 2013;88(3):369–375. 23348083 10.1097/ACM.0b013e318280a6f6

[23] Guy R, Byrne B, Dobos M. Stop think: a simple approach to encourage the self-assessment of learning. Adv Physiol Educ. 2017;41:1. 28188200 10.1152/advan.00174.2016

[24] Gude T, Finset A, Anvik T, et al Do medical students and young physicians assess reliably their self-efficacy regarding communication skills? A prospective study from end of medical school until end of internship. BMC Med Educ. 2017;17:107. 28666440 10.1186/s12909-017-0943-y PMC5493874

[25] Gruppen LD, Garcia J, Grum CM, et al Medical students’ self-assessment accuracy in communication skills. Acad Med. 1997;72(Supplement 1):S57–9. 9347740 10.1097/00001888-199710001-00020

[26] Luria RE The validity and reliability of the visual analogue mood scale. J Psychiatr Res. 1975;12:51–57. 806683 10.1016/0022-3956(75)90020-5

[27] Myford CM Investigating design features of descriptive graphic rating scales. Appl Meas Educ. 2002;15:187–215.

[28] Mohan H, Ryan J, Whelan B, et al The end of the line? The Visual Analogue Scale and verbal numerical rating scale as pain assessment tools in the emergency department. Emerg Med J. 2010;27(5):372–375. 20442167 10.1136/emj.2007.048611

[29] Ismail AK, Abdul Ghafar MA, Shamsuddin NS, et al The assessment of acute pain in pre-hospital care using verbal numerical rating and Visual Analogue Scales. J Emerg Med. 2015;49(3):287–293. 26022936 10.1016/j.jemermed.2015.02.043

[30] Utley R Theory and research for academic nurse educators: application to practice In: Jones, Barlett, editors. Participate in curriculum design and evaluate outcomes-competency 4 (pp. 235–275). Mass USA, London, UK: Publishers International; 2013 Chapter 5. IBSN-13:978-0-7637-7413.

[31] Wewers ME, Lowe NK A critical review of visual analogue scales in the measurement of clinical phenomena. Res Nurs Health. 1990;13:227–236. 2197679 10.1002/nur.4770130405

[32] Lam TCM, Bengo P A comparison of three retrospective self-reporting methods of measuring change in instructional practice. Am J Eval. 2003;24:65–80.

[33] Mattheos N, Nattestad A, Falk-Nilsson E, et al The interactive examination: assessing students’ self-assessment ability. Med Educ. 2004;38(4):378–389. 15025639 10.1046/j.1365-2923.2004.01788.x

[34] Rees C, Students’ SM and assessors’ attitudes towards students’ self-assessment of their personal and professional behaviours. Med Educ. 2005;39:30–39. 15612898 10.1111/j.1365-2929.2004.02030.x

[35] Davis DA, Mazmanian PE, Fordis M, et al Accuracy of physician self-assessment compared with observed measures of competence: a systematic review. JAMA. 2006;296(9):1094–1102. 16954489 10.1001/jama.296.9.1094

[36] Rezler A Self-assessment in problem-based groups. Med Teach. 1989;11:151–156. 2586297 10.3109/01421598909146318

[37] Fitzgerald JT, White CB, Gruppen LD A longitudinal study of self-assessment accuracy. Med Educ. 2002;2003(37):645–649. 10.1046/j.1365-2923.2003.01567.x 12834423

[38] Eva KW, Cunnington JP, Reiter HI, et al How can I know what I don’t know? Poor self assessment in a well-defined domain. Adv Health Sci Educ Theory Pract. 2004;9:211–224. 15316272 10.1023/B:AHSE.0000038209.65714.d4

[39] Billet S Learning through health care wrok: premises, contributions and practices. Med Educ. 2016;50:124–131. 26695472 10.1111/medu.12848

[40] Boud D Enchancing learning through self-assessment. London: Kogan Page; 1995.

[41] Peters S, Clarebout G, Diemers A, et al Enhancing the connection between the classroom and the clinical workplace: A systemic review. Perspect Med Educ. 2016;50:124–131. 10.1007/s40037-017-0338-0 PMC5466563 28293900

[42] O’Connor E, Moore M, Cullen W, et al A qualitative study of undergraduate clerkships in the intensive care unit: it’s a brand new world. Perspect Med Educ. 2017;6(3):173–181. 28390032 10.1007/s40037-017-0349-x PMC5466567

[43] Mann KV, van der Vleuten C, Eva K, et al Tensions inn informed self-assessment: how the desire for feedback and reticence to collect an use it can conflict. Acad Med. 2011;86(9):1120–1127. 21785309 10.1097/ACM.0b013e318226abdd

[44] Sargeant J, Armson H, Chesluk B, et al The processes and dimensions of informed self-assessment: a conceptual model. Acad Med. 2010;85(7):1212–1220. 20375832 10.1097/ACM.0b013e3181d85a4e

[45] Brydges R, Butler D A reflective analysis of medical education research on self-regulation in learning and practice. Med Educ. 2012;46:71–79. 22150198 10.1111/j.1365-2923.2011.04100.x

[46] Schumacher DJ, Englander R, Carracio C Developing the master learner: applying learning theory to the learner, the teacher, and the learning environment. Acad Med. 2013;88(11):1635–1645. 24072107 10.1097/ACM.0b013e3182a6e8f8

